# Herpes Zoster Ophthalmicus Discovered During Pain Consult Possibly Complicated With Dural Sinus Thrombosis and Refractory Headache Managed With Ketamine: A Case Report

**DOI:** 10.7759/cureus.42018

**Published:** 2023-07-17

**Authors:** Laura Furtado Pessoa de Mendonca, Alejandro Hallo-Carrasco, Christian C Prusinski, Christine Hunt

**Affiliations:** 1 Department of Pain Medicine, Mayo Clinic, Jacksonville, USA

**Keywords:** chronic pain management, herpetic neuralgia, ketamine infusion, refractory headache, herpes zoster ophthalmicus

## Abstract

Pain is a common symptom associated with shingles and may precede the onset of the characteristic rash. In the context of herpes zoster ophthalmicus, pain can manifest with severe headaches, posing challenges due to other potentially life-threatening conditions such as stroke and intracranial hypertension. In this report, we present the case of a 51-year-old male with severe headache and imaging findings of dural sinus thrombosis. He was later diagnosed with herpes zoster ophthalmicus and required aggressive inpatient management of neuropathic pain. Despite appropriate treatment, acute herpes zoster can progress to post-herpetic neuralgia, requiring long-term pain management.

## Introduction

Herpes zoster (HZ), or shingles, is caused by the reactivation of the varicella-zoster virus (VZV) that was previously latent in a sensory ganglion after a primary varicella episode. Because of its neurotropic distribution, HZ lesions usually follow dermatomes, and symptoms can be defined as prodromal pain with moderate to severe intensity and shock-like or burning pattern, followed by a vesicular rash in most cases [1]. The ophthalmic branch of the trigeminal nerve (V1) is affected in 10%-20% of shingles cases and is termed herpes zoster ophthalmicus (HZO) [2]. Aside from headache or facial pain, eye tearing and vision loss are also present in HZO [1]. Zoster-related pain can predate the onset of the rash and persist after the rash heals and is classified according to its duration. Acute herpetic neuralgia (AHN) refers to pain that lasts up to 30 days after the diagnosis; subacute herpetic neuralgia (SHN) lasts between one and three months; and post-herpetic neuralgia (PHN) lasts over three months [3,4]. Although many associate HZ with immunosuppression, more than 90% of patients with HZ are immunocompetent [2].

With a prevalence of up to 50%, PHN is the most common complication of shingles, being responsible for a significant decrease in quality of life [4]. Plus, HZO has other specific complications such as corneal damage, uveitis, and life-threatening stroke [2,5]. Pain management during a shingles outbreak is part of the treatment regimen along with antivirals and includes the use of topical agents, anticonvulsants, antidepressants, and, in severe cases, opioids [4,6]. However, this regimen may not be effective in all patients, many of whom go on to suffer debilitating pain [7]. Interventional options may be considered in patients who fail more conservative care. Current interventional pain options are an infusion of N-methyl-D-aspartate (NMDA) antagonists (ketamine), nerve blocks, epidural steroid injections, transcutaneous electrical nerve stimulation, radiofrequency ablation, subcutaneous peripheral nerve stimulation, and spinal cord stimulation [7]. In this case report, we aim to highlight the importance of physical examination for early HZO diagnosis and its complications. We also discuss inpatient management for pain, as well as outpatient treatment for zoster-related pain.

## Case presentation

A 51-year-old male was evaluated in the emergency department (ED) for a new onset of severe, non-positional “headache” that woke him up from sleep. The patient reported that about 36 hours prior to ED evaluation, he had the gradual onset of a left-sided headache increasing in intensity. The headache was localized to the left side of the head (forehead and behind his left eye) and stabbing in nature. The pain was constant, but the intensity of the pain fluctuated from 4/10 to 8/10 on the visual analog score (VAS) with no identifiable exacerbating or alleviating factors. The patient took acetaminophen and ibuprofen at home without improvement. He denied any fevers, chills, vomiting, sinus pressure, cough, vision changes, trauma, or history of migraine headaches before. The patient had a past medical history of alcohol abuse, alcohol-related cardiomyopathy, hypertension, hyperlipidemia, and acute myeloid leukemia in remission. His daily medications at home were fenofibrate, atorvastatin, disulfiram, and metoprolol.

**Figure 1:**
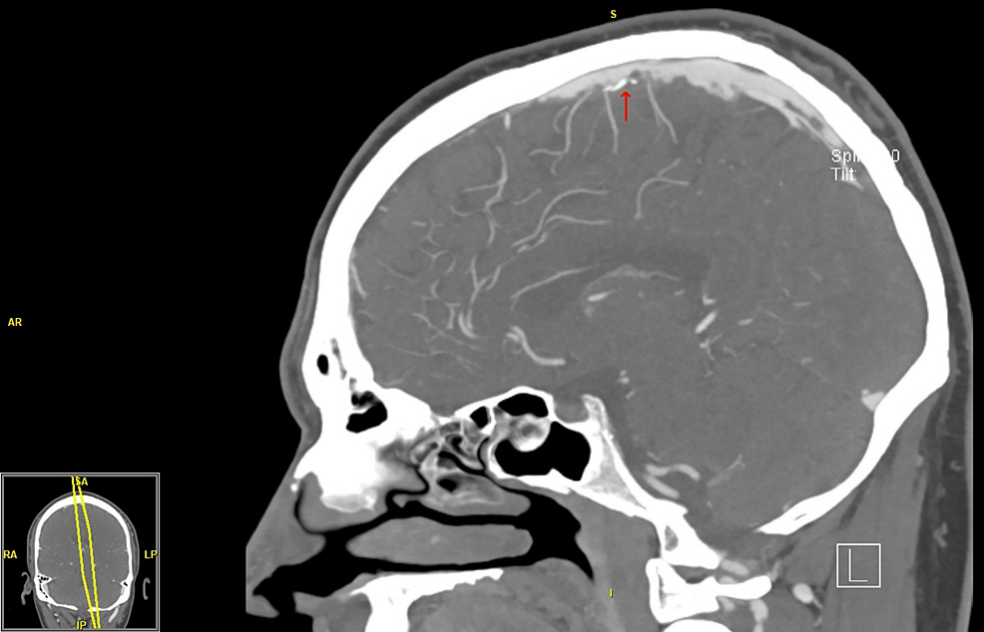
Computed tomography head venogram suggesting dural venous sinus thrombosis (red arrow) AR: anterior right, S: superior, I: inferior, RA: right anterior, SA: superior anterior, LP: left posterior, IP: inferior posterior.

The neurological examination was significant for pain (VAS 5/10), decreased sensation to light touch in the left forehead, and slightly decreased corneal reflex on the left side. Due to the acute presentation and severity, the patient was admitted for further investigation. His computed tomography (CT) head venogram suggested dural venous sinus thrombosis (Figure 1).

Heparin infusion was started, and pain management was initiated with acetaminophen, tramadol, ketorolac, magnesium, diphenhydramine, tapentadol, and morphine, without improvement in pain. Pain Medicine was consulted, and on the fourth day of hospitalization, the Pain Medicine team noted a large erythematous rash extending from the left side of the forehead to the bridge of the nose in the V1 pattern. The patient reported facial numbness with a light touch, and his left eye was visibly irritated, with clear drainage. This was discussed with the primary team (Neurology), and he was then diagnosed with herpes zoster ophthalmicus due to the combination of CT findings and rash distribution. The ophthalmic evaluation showed a faint corneal pseudo dendrite but ruled out corneal necrosis. The pain was not responsive to steroid treatment but was controlled with fentanyl infusion and pregabalin. 

The patient reported worsening pain that was worrisome for possible opioid rebound headaches from the IV medication. Opioids were decreased due to the risk of increased intracranial pressure and oxcarbazepine was added to the therapy, and pregabalin dosage was also increased. His infection was complicated by VZV meningitis, and the patient received prednisone 100 mg daily to decrease inflammation. Procedures were contraindicated acutely during active infection, and the patient refused any interventional procedures for his pain. After nine days of hospitalization, he was discharged to finish his antiviral treatment at home, along with multimodal pain medications (oxcarbazepine, pregabalin, lidocaine cream 4%, and tapentadol).

After three days, the patient returned to the ED complaining of severe pain (VAS 7/10) and was lethargic, but he left the hospital against medical advice. A week after this visit, he returned to the ED complaining of pain 10/10 (VAS), ketamine infusion was started, and he was admitted once again. The infusion rate was initiated at the subanesthetic dose of 0.2 mg/kg/h, then escalated to 0.3 mg/kg/h, and stopped after 48 hours when his pain was controlled (VAS 0/10). The patient's weight was 95.3 kg, and the total amount of ketamine administered during the infusion period was 1.14 g. After 24 hours, the pain returned but was controlled with pregabalin, oxcarbazepine, lidocaine cream 4%, morphine, and amitriptyline. His follow-up CT head venogram no longer suggested dural venous sinus thrombosis, and it was hypothesized that he most likely had developmental septation of the superior sagittal sinus. The patient was discharged after seven days with a similar prescription, but on his follow-up visits, he persisted with pain, despite trials with other drugs such as gabapentin, memantine, and sodium valproate.

## Discussion

We presented a case of a patient diagnosed with HZO during a pain medicine consultation. The Centers for Disease Control (CDC) reports that in the United States, herpes zoster affects approximately one million people each year [8]. Although HZ is commonly associated with elderly and immunocompromised patients, more than 90% of patients with shingles are not immunosuppressed [2]. In fact, conditions such as depression, heart failure, and diabetes have been linked to an increased risk of developing shingles and the use of certain medications, such as statins [1,9]. Our patient’s risk factors included a past medical history of cardiomyopathy, leukemia in remission, and the use of statins.

Our patient reported a stabbing headache that woke him from his sleep. Most patients with shingles will develop a prodrome of dermatomal pain, and in HZO, this will manifest as facial pain or headache. A retrospective study [10] evaluated headache characteristics in patients with HZO, and over 70% reported unilateral, moderate to severe, pre-eruptive stabbing pain. This pain awakened them from sleep in 66.7% of the cases. This calls attention to the importance of considering herpes zoster ophthalmicus as a differential diagnosis in stabbing headaches, even in the absence of rash, after excluding life-threatening hypotheses such as cerebral hemorrhage. Due to the severity of his headache, a CT venogram was performed and initially suggested dural sinus thrombosis, which raises questions about the etiology of his headache: was it secondary to his medical history of cancer or was it a case of herpes zoster vasculopathy? [1]. Nonetheless, this was worrisome because, at that time, the patient received fentanyl injections for his refractory headache which put him at risk of increased intracranial pressure due to the reduction of mean arterial pressure. Given our patient’s medical history of alcohol abuse, it was important to assess the risk for addiction when prescribing opioids.

As previously described, the patient’s rash was consistent with Hutchinson’s sign, which indicates involvement of the nasociliary branch and may predict trigeminal nerve's ophthalmic division (V1 branch) ocular inflammation. Management of shingles’ neuropathic pain often requires a multimodal approach with analgesics, antidepressants, nonsteroid anti-inflammatories, anticonvulsants, opioids, and even sedatives [2,11]. In this case report, our patient persisted with headache refractory from over five different classes of medications, with temporary improvement after a 48-hour infusion of ketamine, an N-methyl-D-aspartate (NMDA) antagonist with strong analgesic properties. Consensus guidelines suggest that infusions for acute pain management should not exceed 1 mg/kg/h, and our patient’s maximum rate was 0.3 mg/kg/h [12]. A meta-analysis has shown that ketamine in the short term is related to statistically significant pain improvement when compared to standard of care, although it may be related to psychedelic effects [13,14]. This reiterates how neuropathic pain can be challenging because pain relief must be balanced with side effects such as sedation and the risk of dependence.

Timing is crucial when diagnosing herpes zoster, and delays in antiviral treatment may increase the risk of developing complications such as post-herpetic neuralgia. Despite our patient being promptly treated when the rash was identified, he still reported headaches more than three months after his diagnosis. It has been demonstrated that for subacute herpetic neuralgia and post-herpetic neuralgia, oral medication alone is not very effective. Many PHN patients refractory to more conservative management may benefit from local anesthetic injections and nerve blocks [15], but given the anatomy of the trigeminal nerve and the high probability of repeating these procedures after the drug is metabolized, long-lasting alternatives should be considered [4].

Peripheral nerve stimulation (PNS) and radiofrequency ablation (RFA) are long-lasting alternatives for subacute herpetic neuralgia and chronic post-herpetic neuralgia [4,16-19]. Studies that have described PNS as a treatment option targeted the supraorbital nerve [4,16-19], while RFA has been utilized to treat the trigeminal ganglion [16,20]. Both PNS and RFA resulted in a statistically relevant reduction in average pain medication dosage, and PNS also showed improvement in quality of sleep. Tao et al. [20] have reported a combination of RFA in the trigeminal ganglion and patient-controlled analgesia with esketamine for HZO, with pain control after two months, but also cautioned readers regarding the potential side effects of this drug such as elevated liver enzymes, dizziness, and lethargy.

Although the studies reported have limitations due to the small sample size, the results are relevant for considering future interventional studies. Whereas zoster is not fully preventable, vaccination can drastically reduce the chances of developing herpes zoster and post-herpetic neuralgia [2,5,8]. In reviewing our patient’s records, he had received only the first dose of the zoster vaccine series.

## Conclusions

Herpes zoster may present with refractory neuralgia that requires management with analgesics, opioids, gabapentinoids, tricyclic antidepressants, anticonvulsants, and anesthetics, such as ketamine. Patients’ comorbidities and past medical history must be considered when prescribing such medications, assessing proper risk for side effects such as sedation and risk of dependence. Even when promptly treated, HZ can be complicated by chronic pain with suboptimal response to medication-only treatment and may benefit from promising neuromodulation techniques provided by pain physicians, reaching long-lasting pain relief.
